# Training School Staff to Prevent Suicide and Promote Mental Health: Outcomes from Virtual PC CARES at School Intervention

**DOI:** 10.1007/s12310-026-09857-3

**Published:** 2026-02-18

**Authors:** Lisa Wexler, Lauren White, Roberta Moto, Tara Schmidt, Justin Heinze, Elizabeth Evans, Angel Zhong, Eleni Kapoulea, Holly Laws

**Affiliations:** 1 School of Social Work, University of Michigan, Ann Arbor, USA; 2 School of Social Work, University of Washington, Seattle, USA; 3 Maniilaq Association, Kotzebue, USA; 4 Institute for Social Research, University of Michigan, Ann Arbor, USA; 5 Department of Health Behavior and Health Equity, University of Michigan, Ann Arbor, MI USA; 6 Department of Psychological and Brain Sciences, University of Massachusetts, Amherst, MA USA; 7 Center for Research on Families, University of Massachusetts, Amherst, USA

**Keywords:** Youth suicide prevention, Rural, Alaska Native, School staff training, Universal mental health promotion

## Abstract

Suicide is a persistent health inequity for rural Alaska Native (AN) youth. School staff in rural AN communities need training and support to prevent suicide and promote mental wellbeing among AN students. This study presents learning and behavior outcomes from Promoting Community Conversations About Research to End Suicide (PC CARES), done virtually in seven monthly, 2 h sessions with school staff. PC CARES brings people together for a series of interactive workshops to gain skills to prevent suicide across the prevention spectrum. Baseline and 1 month follow-up surveys track adult participants’ proximal suicide prevention outcomes of self-efficacy, “community of practice” and behavior outcomes: working together to prevent suicide and promote health; interpersonal support; lethal means reduction and postvention. Mixed effects models tested for pre/post changes for outcomes across participants with complete data (n = 128–140). Moderator analyses tested differential outcomes by number of sessions, cohort, participant role, and school policies. There were 172 PC CARES participants from 21 rural schools and communities in northwestern Alaska in 2020–21 and 2021–22, with average attendance between 4 and 5 sessions. Participants reported significant beneficial changes across all outcomes. Greatest changes occurred for participants with less mental health training (i.e., teachers increased more than school counselors). Greater gains happened in schools where participants reported fewer suicide prevention policies. In rural Alaskan community schools with a high prevalence of suicide, PC CARES At School increased participants’ understanding, collaborations, and actions for supporting AN student mental wellbeing and decreasing suicide risk in culturally responsive ways.

## Introduction

Suicide is a leading cause of death for young people in the United States, and for rural Alaska Native and American Indian (AN/AI) youth, suicide reflects an urgent health inequity. Alaska has the second highest suicide rate in the nation, reflecting the unique geographic, social, and structural challenges its communities face (State of Alaska, 2020). In a recent longitudinal national study, suicide rates among the AN/AI populations consistently increased at an average rate of 3.3% per year (95% CI 3.0, 3.6) throughout the 20 year study period, 1999–2020 (Karaye, 2022). During the COVID-19 pandemic, these disparities intensified; AN/AI youth experienced disproportionate suicide mortality—especially males and 18–24-year-olds—as pandemic-related disruptions to social, cultural, and economic life compounded existing inequities (Bridge et al., 2023). Alaska Youth Risk Behavior Survey (YRBS) from 2019 to 2023 similarly documents persistently elevated rates of suicidal ideation, plans, and attempts among AN youth, including during the pandemic period (Alaska Department of Health, 2024).

Youth suicide rates in rural and remote regions of northwestern Alaska represent one of the most acute public health crises in Alaska and the U.S. We use “rural” and “remote” concomitantly and, at times, interchangeably, to describe the unique geographic and economic characteristics of these communities. “Remote” is a subcategory of rurality that represents regions with the greatest federally classified distance from an urban area (National Center for Education Statistics [NCES], 2024). In Alaska, remote regions are more distinctly defined by being off the road system and accessible only by plane or ferry (Fried et al., 2022). AN teens (ages 15–19) living in remote communities have suicide rates 18 times higher than other teenage Americans (124 versus 6.9 per 100,000) (Wexler et al., 2012). Recent national data further shows that AN male youth (ages 15–24) experience the highest suicide rate (182.6 per 100,000) of any U.S. demographic (182.6 per 100,000), with suicide as the leading cause of death in this group (Craig et al., 2015; State of Alaska, 2020). Among all the Tribal Health regions in Alaska from 2016 to 2019, the Northwest Arctic had the highest per capita rate (118.3 per 100,000) (Alaska Native Tribal Health Consortium [ANTHC] Epidemiology Center, 2021). In Northwest Arctic in 2023, 33.3% of female high school students and 15.9% of male students reported they had seriously considered suicide in the past 12 months (Alaska YRBS, 2025).

### Social Determinants of Alaska Native and American Indian (AN/AI) Youth Suicide

AN/AI suicide is linked to structural violence (Barker et al., 2017; Duran et al., 1998; Evans-Campbell, 2008; Wexler et al., 2024), including historical policies such as the Indian Removal Act of 1830 (Burns et al., 2021), forced boarding schools—which ended in 1972 in Alaska (Hamby et al., 2020; Hirshberg & Sharp, 2005)—and ongoing racism (O’Keefe et al., 2018; Wexler, 2009). Schooling has served as a primary colonizing force whereby institutionalized practices of cultural erasure and forcible assimilation have resulted in marginalization for many AN/AI students within the education system (Washington & Johnson, 2023). The forceful removal and relocation of Indigenous children to government-funded boarding schools throughout the nineteenth and twentieth centuries has contributed to the destruction of Indigenous knowledge systems and linguicide, leading to loss of heritage, fragmentation and disruption of Indigenous kinship networks, and intergenerational trauma (Jacob et al., 2025). Today, the existentially violent legacies of settler colonialism are evident in the education system through the invisibilization of AN/AI history and epistemic privileging of Eurocentric perspectives and Western ways of knowing in school curricula: “Eighty-seven percent of state history standards do not mention Native American history after 1900 and 27 states make no mention of a single Native American in their K-12 curriculum” (National Congress of American Indians [NCAI], 2019, p. 8). Moreover, AN/AI students encounter school-based disciplinary action at disproportionately high rates. While only 1% of the U.S. student population identifies as AN/AI, these students are overrepresented in school arrests and school incidents reported to law enforcement, comprising 2% and 3% of such occurrences respectively (Losen & Martinez, 2020). Thus, AN/AI students are a constituency that has been rendered both *invisible* in terms of contributions and *hyper*-*visible* through disciplinary actions by the U.S. school system in ways that are racialized and historically-constructed.

As such, schools serving primarily AN/AI students are in a unique position to counter this unjust legacy, which can be difficult because of eroded trust between AN/AI families and schools, fragmented services, competing priorities, fiscal constraints, and staffing shortages (Eiraldi et al., 2015). Additionally, mental health safety, services and support are limited (Alaska Mental Health Trust Authority, 2021, 2022). School staff can therefore play a crucial role in creating the conditions for AN/AI students to feel safe and supported, which can increase mental health for all. To reduce the risk of suicide, school staff can also take action to reduce suicide risk when students are struggling and enact postvention activities after a suicide death.

### Upstream Suicide Prevention in Schools

To do this, teachers and administrators can be taught interventions to help them craft culturally safe and respectful support for mental wellbeing and reduce suicide risk among AN/AI students as is part of a broad suicide prevention initiative. Healthy People 2022 (Office of Disease Prevention and Health Promotion [ODPHP], n.d.) and the Institute of Medicine (IOM) promote this broader focus of reducing suicide through universal and selective prevention strategies (van der Feltz-Cornelis et al., 2011) to reach underserved populations that are at elevated risk (Institute of Medicine [US] Committee on Pathophysiology and Prevention of Adolescent and Adult Suicide, 2002). These preventive strategies are enacted “upstream,” *before* someone exhibits a lethal threat to themselves (Berman, 2014).

**Fig. 1:**
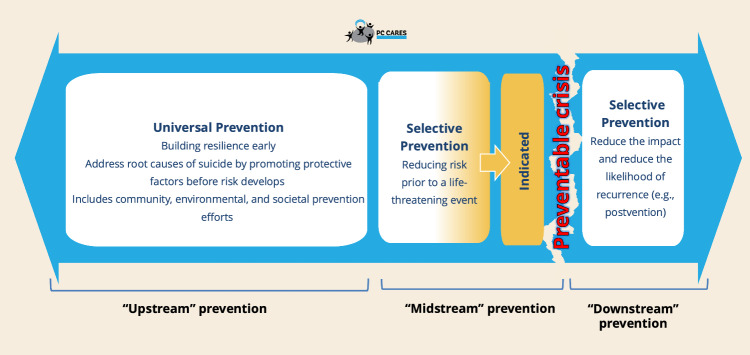
Prevention continuum

“Upstream” or “primary” school suicide prevention strategies are designed to address the root causes of suicide, such as social disconnection or policies that unfairly target different student groups, and promote protective factors like belonging, economic stability, and opportunity (Fig. 1). These approaches are designed to support the conditions for wellbeing, resilience, problem-solving, safety and connection, and are enacted at the population level (rather than individual level) through community-based initiatives, policy changes, and cross-sector partnerships (National Alliance for Suicide Prevention, 2025). In AN/AI communities, upstream suicide prevention approaches that are adapted from existing best practices and built on the cultural and family assets of AN communities can reduce the likelihood of a suicide crisis and death (Cwik et al., 2022; Knipe et al., 2022; Rasmus et al., 2019).

In remote Alaskan communities, availability and access to mental health care providers are often limited (Sawyer et al., 2006; Wang et al., 2005). Over one in five mental health provider positions in rural Alaska are vacant, as compared to one in ten in urban Alaska (Branch, 2014). Cultural barriers to help-seeking was identified as the top cause of suicide in a qualitative study of 35 mostly female Alaska Native participants (Skewes et al., 2023).

Considering the service ecology of these communities and schools, effective suicide prevention must develop *local* capacity and strengthen the social safety net—a multi-level nexus of interrelated protective factors for youth well-being, such as Indigenous kinship networks and robust mechanisms for cultural continuity that promote resilience and community connectedness—addressing the vulnerabilities of persons, institutions and communities to reduce suicide risk before a crisis (Rasmus et al., 2024, 2025). The distinction of local capacity is an important one, since rural schools’ mental health and other services are usually administered regionally by itinerant workers who fly in and out of these remote communities (Saport, 2024). With no continuous presence of “expert” care in the community, reducing suicide risk and enhancing mental wellbeing is in the hands of trusted adults such as parents, teachers, school social workers, Elders, faith leaders, coaches and community health workers (Markowski et al., 2022), many of whom may be the *only* people interacting with vulnerable AN youth (Wexler et al., 2012).

Such universal ‘upstream’ strategies target common interactions and safety precautions that can be enacted in daily life. This approach is broader than more typical “indicated” suicide prevention strategies (Fig. 1) such as Question, Persuade, Refer, (QPR) training, which asks bystanders to refer “at-risk” individuals to services if they observe “warning signs” of suicide (Hangartner et al., 2019; Litteken & Sales, 2018). These signs of elevated risk are rare—in an evaluation of the QPR intervention in Missouri, just 16 of 98 participants in a 2 year follow-up survey had contact with a suicidal individual (Litteken & Sale, 2018).

As an upstream approach in schools, promoting student mental health is increasingly recognized as a critical complement to academic curricula. A recent review of *meta-analyses* reported the consistent positive impact of universal, school-based social and emotional learning programs on a range of student outcomes, including prosocial behavior, academic achievement, and emotional distress (Durlak et al., 2022). When considering mental health-specific programs, universal approaches have demonstrated reductions in anxiety and depression at rates similar to targeted programs, with the advantage of not inadvertently stigmatizing youth selected for more targeted approaches (Feiss et al., 2019). In addition to effects on individual students, school-wide approaches to social-emotional learning, discipline, and school safety can have a positive effect on school climate (Charlton et al., 2021), which in turn is associated with more positive student mental health (Aldridge & McChesney, 2018). Taken together, school wide approaches have consistent, direct, and indirect associations with student mental health. Adopting a universal approach to support suicide prevention that combines culturally responsive framing and input from affected AN/AI communities has the potential to produce similar, if not greater, improvements in student well-being, compared to clinically-based selective and indicated types of interventions.

### PC CARES Intervention Description

As a universal and multilevel prevention strategy, Promoting Community Conversations About Research to End Suicide (PC CARES), brings together teachers, administrators, coaches, classroom aides, parents and others to collaboratively promote mental health, increase environmental and psychological safety, and reduce suicide risk. The focus is on using creative, locally determined approaches that meet the community’s readiness to address suicide (Hofstra et al., 2020; Iskander & Crosby, 2021; Pitman & Caine, 2012; Robinson et al., 2018; Steelesmith et al., 2019; van der Feltz-Cornelis et al., 2011). PC CARES At School offers a series of trainings that build local capacity to prevent youth suicide within schools.

PC CARES is a community-driven health intervention designed to reduce youth suicide by (1) offering supportive adults research-based suicide prevention best practices and (2) strengthening local relationships to build a “community of practice” (CoP) comprised of community members working together with the shared purpose of putting these best practices into action (Wexler et al., 2025). By participating in a series of culturally tailored trainings or “Learning Circles” (LCs), community members learn actionable research-based insights, consider and discuss how the research connects to their local context, personal experiences, and knowledge, and explore ways to apply their learning. Consequently, these sessions build community capacity for suicide prevention and culturally responsive mental health promotion through individual and collective learning and action. This collaborative process fosters a supportive CoP that promotes social connection, enhances support behaviors, and strengthens protective factors related to youth suicide (Wexler et al., 2016). Grounded in principles of cultural responsiveness and community empowerment, PC CARES addresses the unique social determinants of AN youth suicide by leveraging the strengths of the community and fostering sustainable, locally driven prevention efforts (Trout et al., 2018; Wexler, et. al., 2024). PC CARES became registered as Suicide Prevention Best Practice in 2024, a vital next step in expanding the accessibility of the intervention to trusted adults like teachers through professional development avenues like continuing education hours.

### Study Overview

This study presents results from a virtual suicide prevention intervention, PC CARES At School, delivered via synchronous online (Zoom) sessions in partnership with schools and tailored to the conditions experienced during Covid (Wells et al., 2022). The original PC CARES (Wexler et al., 2019) is a community-based intervention that is facilitated in-person by local people trained to host a series of workshops for caring adults in the lives of young people (e.g. coaches, parents, teachers, etc.). The structured and interactive sessions balance evidence-based best practices with local, cultural, and personal knowledges to enable real-time tailoring with the aim of sparking collaborative action on individual, interpersonal, institutional, and community levels.

Here, school staff participated in 7 sessions, or LCs. Each 2–3 h LC focuses on a different topic or topics. Across these sessions, PC CARES At School focused on: (1) Cultural and historical context for Alaska Native youth suicide, (2) Positive youth development and protective factors that reduce suicide risk, 3) Local youth risk behavior survey data, (4) Reflective listening skills practice, (5) The relationship between impulsivity and lethal means safety to prevent suicide, (6) Non-demanding acts of kindness to increase help-seeking, (7) Healing from grief, especially complicated grief involved with suicide, (8) Postvention (decreasing risk of suicide clusters after a suicide death), and (9) Community mobilization. Each session focused on different aspects of suicide prevention—from universal to selective to postvention (after a suicide death)—to increase participants’ everyday tools and build a CoP to take preventative action (Wexler et al., 2016, 2022). Curriculum for PC CARES At School is discussed in detail in Wells et al. (2022).

While each PC CARES LC covers a different topic, they all follow the same pattern. Each LC has an opening and closing; a short information-sharing portion: “What does the research show?” which presents “best practices” and other useful scientific information to spark productive conversations about prevention. The bulk of the session is spent talking and exploring the information, dubbed “What do we think?” and the last section is focused on “What do we want to do?” to prevent suicide and promote youth wellness. This format engages people from different social positions within communities to interpret research evidence and to determine steps needed toward solutions. The sessions are designed to be adaptable by local facilitators so that they fit within cultural and community practices (e.g., involving Elders and blessings, sharing stories, not rushing).

Thus, PC CARES LCs utilize a flexible, engaged educational approach in order to train a variety of people to assist, support, and coordinate with each other to reduce suicide risk and promote the mental wellbeing of students. Sessions are intended for adult community members who are in key positions to identify and build relationships with vulnerable youth (i.e., teachers, school social workers, village-based counselors, coaches, cafeteria workers, health aides, and other health services workers, teachers, school staff). Before the Covid pandemic and this study, PC CARES was always facilitated by someone from the community and in person. During the Covid pandemic, this adaptation—PC CARES At School—was tailored to a rural school context to prepare the school staff to actively reduce suicide risk and to promote mental wellness for the predominately AN students within their schools (Wells et al., 2022) and by fall 2020 was delivered virtually (Cohort 1: October 2020-May 2021 and Cohort 2: August 2021-May 2022). The PC CARES facilitation team included Indigenous* and white^ members (Wexler^, Moto*, McEachern^, Garnie*, Kirk*, Schmidt^, White*) with support from graduate students and staff (Jim Chaliak*, Leanna Isaac*, Megan Leys^, Caroline Bec^). The intervention included six or seven 2 h sessions (“learning circles”) and was implemented synchronously via online video conferencing (Zoom) over six or seven months, depending on the year and schedule of participating schools. Youth under 18 were not invited to participate in the LCs since the focus of the intervention is trusted adults in the lives of young people, particularly school staff.

A new LC was developed for the school-based curriculum focused on postvention planning in schools. These institutional policies and procedures help staff understand their roles and evidence-based strategies for supporting students, staff, and reducing the likelihood of suicide contagion (Poland et al., 2019) if a suicide occurs. This new curriculum expanded the number of LCs and included a self-assessment of each school’s policies and procedures for “what to do” if such a suicide death occurs. According to this self-assessment, over a third (39 of 104) of PC CARES attendees said their school did not have protocols for any of the postvention activities listed, including: (1) Counseling support for students after a suicide occurs; (2) Support staff after a suicide; (3) Assessing students for suicide risk; (4) Providing safe messaging guidelines; (5) Monitoring social media.

In addition, for this new audience of predominantly non-local white school staff, we incorporated shifts towards anti-racist reflexivity into the start of learning circles (Wells et al., 2022). According to a 2010 Alaska Labor Statistics economic trend report, the demand for teachers in Alaska exceeds the supply, and most teachers originate from outside Alaska (Cannon, 2010; Jester et al., 2025). School staff prepared outside Alaska are more likely to leave their position, and turnover among Alaska-trained teachers remains high (Institute of Education Sciences [IES], 2021).

This study examines whether the PC CARES At School intervention made a difference for participants who were mainly school staff. Specifically, it assessed the self-perceived knowledge, self-efficacy, CoP (collaborative relationships for prevention), and reported prevention behaviors and actions before and after the PC CARES intervention (Wexler et al., 2025). In addition, this study examines the effects of moderators on outcomes of interest, including dosage (number of learning circles attended), cohort (which year they attended PC CARES At School), participant characteristics (community and professional roles), and school polices related to suicide prevention and postvention. By simultaneously accounting for these individual and group differences in regression models, the intervention effectiveness can be attributed to either the examined moderators or efficacy of the intervention itself.

### Study Context

Three school districts were involved with PC CARES At School because of community-research partnerships (Wexler et al., 2022) that predated the Covid pandemic. As we describe in other papers, “in 2020, schools closed and tribes restricted travel in and out of the villages, disrupting in-person gatherings. Community members felt the need for suicide prevention programming while trying to manage a public health crisis.” (Evans et al., 2026). The two Tribal health regions have the second and third highest rates of suicide in Alaska, which are 3.3 times and 2.6 times higher than the statewide average (National Institute on Minority Health and Health Disparities [NIMHD], 2025). One of these districts had the highest per capita suicide rate in the state (118.3 per 100,000) (ANTHC, 2021). There, in 2023, 33.3% of female high school students and 15.9% of male students reported they had seriously considered suicide in the past 12 months (Alaska YRBS, 2025). These schools serve two larger regional hubs (pop. 3,000 to 5,000) and 25 remote villages with populations of 300–800 people. Together, the schools have approximately 4300 students, predominantly (75–80%) Iñupiaq and Yup’ik (Alaska Native) (U.S. Census Bureau, 2023). No full time school psychologist is employed in any of these three districts (NCES, 2025), and many of the remote schools have only itinerant social workers and school counselors. Thus, mental health is an important focus of all school staff.

## Methods

### Community-Based Participatory Research

PC CARES is committed to a community-based participatory research (CBPR) framework. Representatives from several villages involved in the research, along with three new members with affiliations to the participating school districts guide the project through participation in the Local Steering Committee (LSC). The inclusion of a LSC reflects best practices in CBPR—supporting shared decision‐making, local leadership, cultural relevance, and sustained partnership (Newman et al., 2011). LSC members help make decisions about study implementation, communication, materials, and activities, and assist with planning and community communications. Members ensure project activities are carried out in ways that are respectful, useful, and aligned with local priorities and cultural values. Monthly video conference meetings and annual in-person meetings are compensated through a yearly stipend for their participation. Members typically serve for a year or more.

### Authors’ Positionality Statement

The authors of this paper include the principal investigator (Wexler), co-developers and facilitators of the curriculum (Moto), a school-based intervention expert (Heinze), PC CARES staff (Schmidt, White, Zhong), and researchers (Evans, Laws, Kapoulea). The research team comprises a diverse and intergenerational group of Native and non-Native researchers, community practitioners, and experts based in Michigan and Alaska, many of whom have been in long-term research partnerships, and all who strive to center decoloniality, cultural humility, and reflexivity.

### Ethics Approval

This study received ethical approval from the Alaska Area IRB for regions that rely on them for oversight, and regional Tribal ethics boards in the areas of Alaska organized by tribal health organizations before its delivery in participating communities. Engaging local community members, institutional partners, and Tribal IRBs reflects our commitment to respectful and reciprocal research partnerships based in communities. All ethical procedures, including informed consent, were done at a regional and individual participant level.

### Recruitment

Recruitment differed slightly for each year of the intervention and reflects the partnering school districts annual priorities and availability. These procedures included offering all-staff training and “opt in” staff training, emails, and short presentations at staff meetings for health and social service organizations and school districts.

#### Intervention Recruitment

Recruitment began with an educational outreach session with school and Tribal behavioral health staff early in the school year (October 2020 and August 2021). These sessions were held virtually and varied across districts from a 20 min overview to a 2 h staff workshop focused on suicide prevention. Next, participants were invited to participate in PC CARES learning circles as part of a free 3-credit university course or as a non-credit-seeking participant. To receive university course credit, participants had to attend a minimum of 4 of the 6–7 sessions. These credits qualified for maintenance of teachers’ Alaska accreditation and fulfilled the requirements for suicide prevention education required by the state every 2 years. Credit was granted on a pass/fail basis; however, if attendance was not sufficient to grant credit (4 or more sessions), participants were given the option to withdraw, rather than fail, the course. In the second year, short writing reflection assignments were added to the course. Social workers, counselors, and health aides (Alaska Community Health Aide Program, n.d.) who participated in PC CARES sessions could also receive continuing education hours. In some school districts, learning circles were scheduled to occur during regular staff professional development time.

Participants (n = 167) from 21 different communities within the Tribal health regions elected to participate in the intervention.

#### Survey Recruitment

Participants were invited to complete Steps to Prevention (StP) Surveys before their first session (Baseline) and after the last session (Follow-Up). Surveys were distributed via email approximately one month after the last session. Participants who didn’t respond were sent email reminders every 3–5 days for two weeks to boost participation. Survey incentives were $20 online gift cards to a major retailer for the Baseline survey, and $40 for the follow-up survey.

### Measures and Analyses

All statistical calculations were performed using SPSS Version 26 (IBM Corp., 2019).

#### Measures

##### PC CARES Steps to Prevention (StP) Survey

The PC CARES Steps to Prevention (StP) Surveys were used in previous evaluations of PC CARES (Markowski et al., 2022; Wexler et al., 2019; White et al., 2022), and assess participants’ learning outcomes: suicide prevention self-efficacy (3 items), wellness self-efficacy (3 items), community of practice (collaborative relationships; 3 items), as well as behavioral outcomes: working together to prevent suicide and promote health (5 items); interpersonal support giving (7 items); and postvention actions (5 items). Each learning outcome reflects a different type of learning ranging from what participants know, others on how confident they feel in applying skills, the support they give or receive, or the actions they take to prevent suicide and promote wellness. Although related, these outcomes measure separate constructs, allowing the survey to capture distinct areas of growth, as shown in our prior work (Wexler, et al., 2025). StP survey was administered to participants at Time 1 (pre) and Time 2 (post) about 6 months apart. Items from each continuous subscale were averaged to create a total score, and binary items indicating presence or absence of a behavior were summed to create behavioral measures. Paired sample t-tests were conducted to compare pre and post scores (Table 1), similar to previous analyses of the StP measure (Wexler et al., 2019). Reliability analyses indicated that all learning outcome subscales had adequate to excellent internal reliability (Cronbach’s alpha > 0.75) at both timepoints, while behavior scales had poorer, but acceptable, reliability.

**Table 1:** PC CARES steps to prevention (StP) measure

Item	*N*	Mean (SD)		*t*	*p*	Cronbach’s α	
		Pre	Post			Pre	Post
Suicide Prevention Self-Efficacy*	133	**5.23 (1.19)**	**6.17 (0.71)**	**7.09**	** < 0.001**	0.856	0.837
I know how to talk safely about suicide, in ways that help with prevention							
I know how to decrease suicide risk for others by the way I talk about suicide							
I feel confident that I can do things to prevent suicide							
Wellness Self-Efficacy*	133	**5.79 (1.07)**	**6.30 (0.74)**	**4.18**	** < 0.001**	0.652	0.758
There are things I can do to promote wellness here							
I talked about how to create a healthy environment for youth as they grow up							
I know how I can make positive changes for school wellness							
Community of Practice*	133	**5.32 (1.16)**	**5.73 (1.11)**	**2.79**	**0.006**	0.679	0.753
Many people in this community work together for suicide prevention/wellness							
I have regular opportunities to work with others to increase wellness							
I have many people to work with in my community to prevent suicide							
Work Together to Prevent Suicide and Promote Health Behaviors**	128	**3.05 (1.82)**	**3.94 (1.71)**	**4.08**	** < 0.001**	0.737	0.711
I asked someone for help doing prevention/wellness work when I needed it							
I spoke up about what the school can do to reduce the risk of youth suicide							
I talked with others about wellness							
I worked with others to increase wellness in the school community							
I worked with others to prevent suicide							
I let others know what resources are available for prevention							
Interpersonal Support Behaviors**	128	**1.74 (1.59)**	**3.00 (1.48)**	**3.91**	** < 0.001**	0.719	0.660
I spent time listening to a teen or child who just wanted to talk about their experience							
I trusted others in the school community to hear what I have to say							
I reached out to a child or teen who was hurting (alone, sad, angry)							
I helped a youth (child/teen) who was down get help (Behavioral Health Services, Alaska Careline, etc.)							
I reminded someone that just listening to someone can be more supportive than giving advice							
I quietly listened to a youth (child/teen) who had a problem, reflecting back to them what I heard							
I encouraged others to offer small acts of kindness when someone was having a hard time							
Postvention Behaviors**	128	**1.74 (1.59)**	**3.00 (1.48)**	**6.27**	** < 0.001**	0.620	0.566
I shared only the basic facts of a suicide (avoiding details)							
I spoke to someone about how to talk safely after a suicide							
I talked about how suicide is no one’s fault							
I shared that it can be harmful to honor someone who died by suicide more than is done for other deaths							
I talked about how we can help prevent further harm after a suicide happens							

*7-point Likert agreement scale, **Yes/No

##### New Baseline School Policy Measure

Because PC CARES was being delivered mainly to school staff in 21 different communities, it was important to capture each institution’s current suicide prevention policies and “readiness” (Cox et al., 2016; Owens, 2014; Richter et al., 2022) at the start of the intervention to see how context—including school policies—impacts the readiness of participants to act on what they have learned (moderating outcomes). A new measure of baseline school-level policy aims to capture participants’ knowledge and perceptions of the training and support already happening in each school related to suicide prevention and postvention (Table 2). Based on our hypothesized theories of the school policy constructs, we conducted a confirmatory factor analysis. During confirmatory factor analysis, comparing three- and two-component solutions, the two-component solution with prevention policy and postvention policy was the most meaningful (Edwards et al., 2000). One factor aligned closely with items related to prevention policy [e.g., “*My school provides adequate resources (e.g., training, staff time for planning, support”) to develop and maintain suicide safety plans*], while the other was consistent with postvention policy (e.g., “*I am aware of my school’s protocols if a suicide death affects my school”*). We reduced the number of items by excluding five items with component loadings < 0.7. The Cronbach’s coefficient < was above 0.8 for each of the two components, thus confirming a good internal consistency between the items within the construct (Table 2). Mean scores for each of the constructs were calculated for use in subsequent analyses. We performed a principal component analysis (PCA) with oblique (oblimin) rotation items collected at Time 1 (Suhr, 2005). To test the appropriateness of the data for PCA, we tested the underlying assumptions using the Kaiser–Meyer–Olkin index (KMO) of sampling adequacy. Results were > 0.7, indicating that patterns of correlations are relatively compact and suitable for PCA. According to Bartlett’s sphericity test (χ^2^ = 2116.90, *df* = 28, *p* < .001), multicollinearity and singularity were not violated, indicating that the independent variables are not correlated with each other, and that a PCA would verify a data reduction technique can compress the data in a meaningful way. The pattern matrix of a two-component solution appeared to be the most meaningful and interpretable, and accounted for 79.73% of the total variance (Tables 3, 4).

**Table 2:** New baseline school policy measure**—**pattern matrix and Cronbach's coefficient α for policy constructs

	Item	Factor loading	Cronbach’s α
Prevention Policy*	My school’s leaders are committed to actively working on suicide safety at my school	0.806	0.886
	My school provides adequate training and support for school personnel to respond to suicide death or attempts affecting our school	0.917	
	I am aware of my school’s protocols if a suicide attempt affects my school	0.878	
	I know what my roles and responsibilities are if a suicide attempt affects my school	0.829	
Postvention Policy*	My school currently has clear and effective suicide policies for postvention	0.861	0.851
	I am part of a team that is working to improve suicide safety at my school	0.736	
	I am aware of my school’s protocols if a suicide death affects my school	0.911	
	I know what my roles and responsibilities are if a suicide death affects my school	0.856	

^*^7-point Likert agreement scale

**Table 3:** Paired participant racial/ethnic demographics, all regions and cohorts combined

Race/Ethnicity	Count	Proportion (%)
White	78	47.3
Race unknown	39	23.6
Alaska Native/American Indian	18	10.9
Asian	11	6.7
More than one race	8	4.8
Hispanic/Latino	6	3.6
Black/African American	5	3.0
*Gender*		
Female	127	77.0
Male	37	22.4
Other	1	0.6
*Role*		
Teacher	86	52.1
Missing	22	13.3
School Administrator	16	9.7
Other Not Listed	11	6.7
Other Tribal Health Employee	10	6.0
Therapist	9	5.5
Classroom Aide	5	3.0
Village-Based Counselor/Behavioral Health Aide	5	3.0
Other School Staff (Cafeteria Worker/Janitorial Staff/Front Desk)	1	0.6
Total	165	100

**Table 4:** Attendance summary

School Year	Attended 7 LCs	Attended 6 LCs	Attended 5 LCs	Attended 4 LCs	Attended 3 or fewer LCs	Total attendees
2020–21	–	40	17	12	15	84
2021–22	9	15	15	15	29	83

#### Mixed Effects Regression Models

To estimate the relationship of moderators of interest with changes on the Suicide Prevention Self-Efficacy (sePrev), Wellness Self-Efficacy (seWell), Community of Practice (CoP), Work Together to Prevent Suicide and Promote Health Behaviors (WorkTog), Interpersonal Support Behaviors (IntSupp), and Postvention Behaviors subscales, we used mixed effects regression models using the SPSS MIXED procedure (Peugh & Enders, 2005). This approach allows for the nesting of repeated measures (Level 1) within individuals (Level 2) within communities/schools (Level 3). First, we created a model estimating the change in each outcome over time (pre- to post-). The main difference between these models and the paired samples t-tests is that these models employed maximum likelihood estimation rather than listwise deletion, and the analyses properly accounted for the nesting of individuals within distinct communities/schools, which induces interdependence in scores not accounted for by paired tests.

##### Moderator Models

Next, we tested moderators to see whether changes in the outcomes differed depending on participant differences (See Table 5):

1. *No moderators*
2. *Number of learning circles (LCs;* < *4 LCs is reference)*—A dose–response relationship between the number of learning circles attended and the diversity, frequency, and number of prevention-related actions has been found in previous PC CARES research, so it is hypothesized that higher attendance would be associated with positive outcomes (Wexler et al., 2019). Attendance of at least 4 LCs was required to “pass” for college credit and was enough participation in the past to be associated with significant outcomes, thus we selected 4 as reference.
3. *Cohort (2 is reference)*—There were key differences between each cohort that were likely to produce differences in outcomes: varied recruitment strategies, previous exposure to PC CARES At School (“contamination” or spread of knowledge and behaviors from the previous cohort), the context of the pandemic itself (e.g., school shutdowns in cohort 1 and vaccine availability in cohort 2), and because of the earlier start, we were able to offer an additional learning circle for cohort 2 which focused on principles and practices of community change.
4. *School-based role (teacher is reference)*—Understanding how school staff with varied roles and experience within a school, school district, or community react to the PC CARES At School intervention will show where the program can be most effective.
5. *School prevention policy*—We included survey items about the level of existing policies and programs for suicide prevention, and
6. *School postvention policy*—PC CARES At School was hypothesized to have an amplifying effect on schools with existing institutional support for both prevention and postvention.

**Table 5:** Moderators of change in in *Self Perceptions* of suicide prevention self-efficacy and wellness self-efficacy: Mixed Effects Models 1–6 Results

	sePrev				seWell		
		*Est*	*SE*	*p*	*Est*	*SE*	*p*
*Model 1: No moderator*							
Intercept		5.22	0.10	< 0.001	5.80	0.95	< 0.001
Linear Change		**0.93**	**0.09**	** < 0.001**	**0.50**	**0.09**	** < 0.001**
*Model 2: # of LCs*							
Intercept		5.20	0.22	< 0.001	5.90	0.20	< 0.001
Linear Change		0.77	0.22	0.001	0.27	0.21	0.212
Int*#LCs		0.03	0.25	0.915	−0.13	0.22	0.554
Change*#LCs		0.18	0.24	0.451	0.29	0.23	0.222
*Model 3: Cohort*^a^							
Intercept		5.42	0.14	< 0.001	5.19	0.13	< 0.001
Linear Change		0.64	0.13	< 0.001	0.29	0.13	0.026
Int*Cohort 1		−0.39	0.20	0.051	−0.23	0.18	0.213
Change*Cohort 1		**0.55**	**0.18**	**0.003**	**0.40**	**0.18**	**0.026**
*Model 4: Role*^b^							
Intercept		5.10	0.13	< 0.001	5.80	0.12	< 0.001
Linear Change		1.05	0.12	< 0.001	0.53	0.11	< 0.001
Int*Therapist		0.45	0.28	0.115	−0.12	0.25	0.631
Int*School Administrator		0.21	0.32	0.515	0.20	0.28	0.476
Int*Staff		0.82	0.53	0.125	0.45	0.47	0.342
Change*Therapist		−0.21	0.27	0.446	0.20	0.25	0.439
Change*School Administrator		−0.33	0.31	0.283	−0.08	0.28	0.774
Change*Staff		−0.98	0.50	0.056	−0.56	0.46	0.229
*Model 5: School Prevention Policy*							
Intercept		5.22	0.11	< 0.001	5.76	0.10	< 0.001
Linear Change		0.97	0.10	< 0.001	0.54	0.10	< 0.001
Prevention		0.31	0.15	0.041	0.21	0.14	0.138
Change*Prevention Policy		**−0.37**	**0.14**	**0.011**	−0.22	0.14	0.102
*Model 6: School Postvention Policy*							
Intercept		5.21	0.11	< 0.001	5.75	0.10	< 0.001
Linear Change		0.97	0.10	< 0.001	0.54	0.10	< 0.001
Postvention		0.18	0.14	0.202	0.03	0.13	0.792
Change*Postvention		−0.14	0.13	0.286	0.01	0.13	00.931

sePrev = suicide prevention self-efficacy. seWell = wellness self-efficacy. LCs = learning circles. ^a^Reference = Cohort 2. ^b^Reference = Teacher

Bold indicates significant change from Pre- to Post-Treatment or significant moderation of Pre-Post change

#### Post-Hoc Analyses

After reviewing our initial results, we conducted exploratory models to determine if significant cohort effects were partially or fully explained by, or collinear with, participants’ roles. That is, for outcomes with significant Cohort x Change interaction effects (i.e., Knowledge, Support, sePrev, seWell, and Postvention Behaviors), we tested models that regressed each outcome on: (1) linear change, (2) role, (3) cohort, and the (4) cohort x change interaction.

### Findings

#### Participants

Participating school staff and community members represented three rural school districts residing in two Tribal health regions, encompassing 21 communities across a geographic area in northwestern Alaska the size of Oregon and Washington combined. Adult participants reflected diverse racial and ethnic backgrounds, with most identifying as white (47.3%), followed by race unknown (23.6%), Alaska Native/American Indian (10.9%), and Asian (6.7%). Most participants were female (77%), and just over half were teachers (52%) with school administrators, classroom aids, behavioral health and Tribal health employees making up most of the rest.

In the first year, 62 out of 84 (74%) attendees in the 2020–21 school year received college credit for their participation in PC CARES. In the second year of the intervention, 55 of 83 attendees (66%) received college credit for their participation in the class.

#### Attendance

In the first cohort, 69 out 84 participants (82%) attended at least 4 out of 6 learning circles. In the second cohort, attendance was less consistent, with 54 out of 83 (65%) participants attending 4 or more out of 7 sessions. Only three people participated in the intervention without receiving credit in the first cohort, two of whom attended all six sessions. In the second cohort, 12 participants attended four or more sessions without receiving college credit. Overall, 70% of attendees received college credit, 7% received social work contact hours, and 4% received no educational credit benefit. Session attendance is included in Table 4.

#### Response Rate

Participant attrition to follow-up varies depending on the outcome. Focusing on the most reported outcome, Wellness Self-Efficacy, a total of 143 out of 167 (85%) PC CARES participants filled out surveys, 96 with both timepoints, 33 baseline only, 14 with followup but no baseline. Missing data on the repeated measures outcome variables were handled using Full Information Maximum Likelihood Estimation (FIML), a modeling-based correction for missingness that is equivalent in its assumptions of missingness patterns to most applications of multiple imputation, and is considered less problematic than listwise deletion (Allison, 2001; Shin et al., 2017). The FIML estimation technique used in the mixed effects regression models allows anyone with at least one valid outcome at either timepoint to stay in the model.

#### Mixed Effects Regression Models

Paired samples *t-*tests conducted as preliminary tests of outcome change demonstrated significant change in each of the hypothesized outcomes (Table 1). Mixed effects regression models similarly demonstrated significant positive change when comparing changes from pre- to post-scores on all outcomes. Moderators were consistent with hypotheses, but not uniformly so (Tables 5, 6, 7).

**Table 6:** Moderators of change in community of practice (CoP), work together to prevent suicide and promote health, interpersonal support behavior, and postvention behaviors: mixed effects models 1–3 results

	CoP			WorkTog			IntSupp			Postvention behaviors		
	*Est*	*SE*	*p*	*Est*	*SE*	*p*	*Est*	*SE*	*p*	*Est*	*SE*	*p*
*Model 1: No moderator*												
Intercept	5.34	0.11	< 0.001	3.12	0.19	< 0.001	4.83	0.18	< 0.001	1.77	0.16	< 0.001
Linear Change	**0.36**	**0.11**	**0.002**	**0.88**	**0.18**	** < 0.001**	**0.91**	**0.17**	** < 0.001**	**1.28**	**0.16**	** < 0.001**
*Model 2: # of LCs*												
Intercept	5.34	0.22	< 0.001	3.21	0.35	< 0.001	5.23	0.35	< 0.001	2.23	0.30	< 0.001
Linear Change	0.32	0.28	0.258	1.29	0.44	0.004	0.79	0.43	0.067	0.78	0.39	0.049
Int*#LCs	0.00	0.24	0.989	−0.13	0.38	0.732	−0.51	0.39	0.192	−0.60	0.33	0.073
Change*#LCs	0.05	0.30	0.872	−0.47	0.48	0.333	0.17	0.47	0.724	0.62	0.43	0.153
*Model 3: Cohort*^a^												
Intercept	5.50	0.15	< 0.001	3.40	0.25	< 0.001	5.05	0.25	< 0.001	2.16	0.22	< 0.001
Linear Change	0.13	0.17	0.426	**0.91**	**0.26**	** < 0.001**	**0.63**	**0.25**	**0.015**	**0.94**	**0.23**	** < 0.001**
Int*Cohort 1	−0.33	0.20	0.106	−0.56	0.32	0.081	−0.43	0.33	0.188	−0.77	0.28	0.006
Change*Coh 1	0.42	0.22	0.064	−0.02	0.35	0.967	0.52	0.34	0.134	**0.66**	**0.31**	**0.036**

CoP = community of practice. WorkTog = working together to prevent suicide and promote health. IntSupp = interpersonal support behaviors. LCs = learning circles. LCs = learning circles. ^a^Reference = Cohort 2

Bold indicates significant change from Pre- to Post-Treatment or significant moderation of Pre-Post change

**Table 7:** Moderators of change in community of practice (CoP), work together to prevent suicide and promote health, interpersonal support behavior, and postvention behaviors: mixed effects models 4–6 results

	CoP			WorkTog			IntSupp			Postvention behaviors		
	*Est*	*SE*	*p*	*Est*	*SE*	*p*	*Est*	*SE*	*p*	*Est*	*SE*	*p*
*Model 4: Role*^a^												
Intercept	5.25	0.14	< 0.001	2.91	0.23	< 0.001	4.58	0.22	< 0.001	1.53	0.20	< 0.001
Linear Change	**0.45**	**0.14**	**0.001**	**0.83**	**0.22**	** < .001**	**1.20**	**0.21**	** < .001**	**1.49**	**0.20**	** < .001**
Int*Therapist	0.25	0.28	0.388	0.63	0.44	0.159	0.83	0.45	0.070	0.57	0.38	0.137
Int*School Administrator	0.13	0.32	0.672	0.65	0.50	0.191	0.12	0.51	0.811	0.47	0.43	0.272
Int*Staff	0.38	0.52	0.467	−0.49	0.81	0.551	0.36	0.83	0.670	0.27	0.70	0.696
Change*Therapist	−0.20	0.33	0.541	−0.21	0.54	0.697	**−1.39**	**0.49**	**0.006**	−0.39	0.47	0.413
Change*School Administrator	0.04	0.37	0.918	0.57	0.57	0.324	0.19	0.53	0.715	−0.45	0.51	0.372
Change*Staff	−0.29	0.59	0.630	1.26	1.08	0.247	−0.84	1.00	0.401	−1.39	0.95	0.144
*Model 5: School Prevention Policy*												
Intercept	5.35	0.12	< 0.001	3.08	0.20	< 0.001	4.82	0.19	< 0.001	1.72	0.16	< 0.001
Linear Change	0.39	0.12	0.001	0.88	0.19	< 0.001	0.93	0.16	< 0.001	1.38	0.17	< 0.001
Prevention	0.08	0.17	0.620	0.01	0.27	0.985	0.05	0.27	0.837	−0.04	0.22	0.865
Change*Prevention	−0.03	0.17	0.872	−0.18	0.27	0.501	−0.25	0.23	0.270	−0.11	0.23	0.636
*Model 6: School Postvention Policy*												
Intercept	5.34	0.11	< 0.001	3.07	0.19	< 0.001	4.81	0.18	< 0.001	1.72	0.16	< 0.001
Linear Change	0.39	0.12	0.002	0.87	0.19	< 0.001	0.91	0.19	< 0.001	1.38	0.17	< 0.001
Postvention	0.19	0.14	0.199	0.22	0.24	0.368	0.30	0.23	0.194	0.11	0.20	0.605
Change*Postvention	0.14	0.15	0.375	−0.05	0.25	0.837	−0.20	0.24	0.393	−0.24	0.21	0.257

CoP = community of practice. WorkTog = working together to prevent suicide and promote health. IntSupp = interpersonal support behaviors. LCs = learning circles. LCs = learning circles. ^a^Reference = Teacher

Bold indicates significant change from Pre- to Post-Treatment or significant moderation of Pre-Post change

##### Model 1: Three Level Change Models: No Moderators

Scores for all outcome variables significantly increased from pre to post (*p* < 0.001), representing greater knowledge, more confidence (self-efficacy) in their suicide prevention and wellness promotion skills, greater support for helping students at risk, after an attempt, or after a suicide death, and more collaborative relationships among PC CARES participants. Proximal outcomes related to self-perceptions all changed in a positive direction from pre- to post-PC CARES At School intervention, including: Suicide Prevention Self-Efficacy, Wellness Self-Efficacy, and Community of Practice (Table 5), as well as the behavioral outcomes of Work Together to Prevent Suicide and Promote Health, Interpersonal Support Behavior, and Postvention Behaviors (Table 6).

##### Model 2: Number of Learning Circles (LCs)

There were no significant differences in outcome change between participants who attended at least four LCs versus those who attended fewer than four LCs. See Table 5 for relevant statistics.

##### Model 3: Cohort

**Fig. 2:**
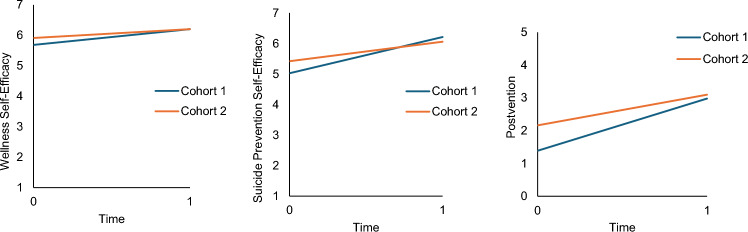
Cohort moderates changes in wellness self-efficacy, prevention self-efficacy, and Postvention behaviors. *Note.* Change slopes in these three outcomes were significantly stronger (more positive) in Cohort 1 compared with Cohort 2

Cohort 1 demonstrated a significantly greater improvement in self-perceptions of Suicide Prevention Self-Efficacy (*b* = 0.55, *SE* = 0.18, *p* = 0.003), Wellness Self-Efficacy (*b* = 0.40, *SE* = 0.18, *p* = 0.026), (Table 5) and Postvention Behavior (*b* = 0.66, *SE* = 0.31, *p* = 0.036; Table 5) scores over time than Cohort 2 (Fig. 2).

##### Model 4: Role

**Fig. 3:**
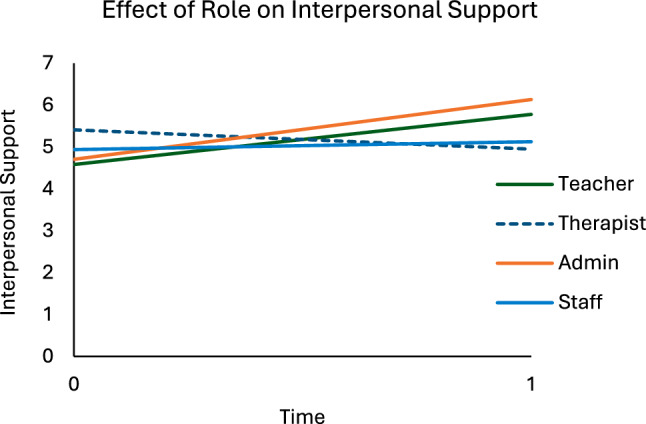
Role moderates interpersonal support giving. *Note.* Change slopes this outcome was significantly weaker (less positive) in therapists compared with teachers

Interpersonal Support behavior scores for teachers significantly increased from pre- to post (*b* = 1.20, *SE* = 0.21, *p* < 0.001). Therapists’ interpersonal support change scores were effectively flat, and significantly different from the positive change in teachers (*b* = −1.39, *SE* = 0.49, *p* = 0.006; Table 7) Thus, therapists’ interpersonal support scores changed significantly less than teachers’ scores (Fig. 3).

##### Model 5: School Prevention Policy

**Fig. 4:**
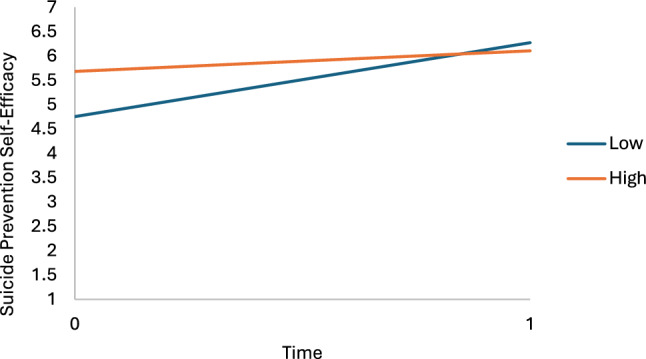
School policies about suicide prevention moderate self-efficacy change. *Note.* Change slopes this outcome was significantly stronger (more positive) in those from schools with lower rated policies

Individuals with lower School Suicide Prevention Policy (i.e. less suicide prevention training and support) at baseline showed a significantly stronger increase in Suicide Prevention Self-Efficacy (*b* = −0.37, *SE* = 0.14, *p* = 0.011) scores over time (Fig. 4; Table 5). School Suicide Prevention Policies did not moderate any other outcomes.

##### Model 6: School Postvention Policy

Baseline School Postvention Policy scores, (i.e. training and support for what to do after a suicide occurs) showed no significant association with change in any outcome. (See Tables 6, 7).

##### Follow-up Analyses for Cohort Effects

We observed that the makeup of participant roles differed for each cohort, with more non-school staff comprising the second cohort. To determine if significant cohort effects were partially or fully explained by participants’ roles, we tested model parameters for mixed effects regression models. After controlling for role, the interaction of Cohort x Change remained significant for Suicide Prevention Self Efficacy (*b* = 0.57, *SE* = 0.19, *p* = 0.004), and Postvention Behaviors (*b* = 0.76, *SE* = 0.33, *p* = 0.023). Regarding Wellness Self Efficacy, the interaction effect of Cohort x Change was no longer significant (*b* = 0.35, *SE* = 0.18, *p* = 0.056) after controlling for role. This means that the rate of change in self-perception of wellness self-efficacy for each cohort was not significant when accounting for participants’ role (e.g., teacher, therapist, etc.) For all models, the main effects of role were not significant (*p* > 0.05) (Table 5).

## Discussion

In a new format (virtual) and in a different context (school), PC CARES At School participants reported significant beneficial changes across all reported learning and behavioral outcomes. This result is particularly hopeful when considering that this intervention was delivered virtually and primarily with school staff for the first time, across 21 different communities, with varying degrees of participation from site to site. Previous PC CARES trials were successful in remote and rural communities with predominantly AN participants and showed similar results in changes in participants’ learning and behavior outcomes (Wexler et al., 2019). Here, our analysis shows that participants built communities of practice, and increased their wellness and suicide prevention self-efficacy (Wexler et al., 2025) and, importantly, increased their prevention-oriented behaviors. The behavior change reported includes interpersonal support, suicide prevention, and mental health promotion actions taken with others (“Working Together” scale), lethal means reduction and planning or actions taken to reduce harm after a suicide occurs (postvention).

A well-established body of literature supports the efficacy of approaches that target school staff to promote positive student mental health by altering the school environment through community relationship building, staff training, and procedural, programmatic and physical changes to schools’ structures (O’Reilly et al., 2018). Although many such approaches are universal in that they engage all students and staff within schools, there are few interventions specifically tailored to Alaska Native (AN) student populations, who experience a disproportionately high burden of mental health injury. These inequities are compounded by the fact that many AN students attend schools in rural locations, which face additional challenges associated with culturally-responsive youth mental health services (Owens et al., 2013) and a need for more approaches tailored to youth in rural settings (Berryhill et al., 2022).

The virtual intervention included six or seven sessions (2 h each; monthly; virtual, synchronous delivery) designed to improve teachers’, administrators’ and therapists/counselors’ capacity to promote mental health and prevent suicide. Clauss-Ehlers and Garagiola (2023) note the importance of fostering interdisciplinary connections as well as school-community connections to support school mental health outcomes. In this way, PC CARES activated existing resources and strategically supported the increase of school staff’s capacity to address mental wellness and suicide. Building local capacity to work together can be a steppingstone for further positive change, such as the development of peer support programs, co-developing culturally responsive suicide prevention strategies, integrating interdisciplinary support systems into everyday school practices, supporting partnerships between schools, families, and the community.

PC CARES may represent an effective and feasible community-level strategy for mitigating the impacts of the national behavioral health workforce shortage. As of December 2024, 122 million Americans live in federally designated mental health professional shortage areas (Health Resources and Services Administration [HRSA], 2025), with these shortages disproportionately affecting rural regions and marginalized communities (Counts, 2023; Substance Abuse and Mental Health Services Administration [SAMHSA], 2024). Current efforts to address these shortages focus on increasing the number of mental health providers through recruitment, training, and pipeline programs (Giambrone & Thomas, 2024). However, increasing the number of mental health professionals can take extensive time, infrastructure, and resources. For youth specifically, expanding the capacity of the school workforce and local community supports (e.g., family and Elders) to address student mental health could be a more feasible path. A recent convening of federal and state policymakers, school district leaders, researchers, and national organizations recommended investing in the mental health literacy of staff to advance comprehensive school mental health systems: “Equipping educators with social and emotional skills and mental health literacy will prepare them to best support student mental health and create a healthier workforce” (Hoover et al., 2019, p. 20). PC CARES At School built on the preexisting skillsets of school staff and community members to expand the scope of their supportive practices for youth, thus enabling them to better take local action to address mental wellness and reduce suicide risk without substantial additional strain on school resources. It also aligned knowledge for school staff across diverse roles, creating more shared understanding of effective suicide prevention and fostering greater collaboration. All roles except therapists (n = 9)—who have comparatively more experience in supporting mental health—showed increases in interpersonal support-giving after PC CARES.

Behavioral outcomes included more collaborations among school staff and community members. The PC CARES model builds on the idea of developing a Community of Practice (CoP) to support ongoing collaborations and problem-solving as an important and sustainable way to increase mental health and wellness. When rural school staff, who are usually not from the same culture or area as rural AN students, connect with local community members to discuss local data and perspectives on wellness, suicide prevention knowledge is placed in a historical and cultural context, highlighting unique local challenges and strengths. The adaptation to an online format included reminders about white dominant culture and a pivot to “something else” (Okun, 2019; Wells et al., 2022). These reminders targeted culturally misaligned norms embedded in white dominant culture, such as “Perfectionism” (“Mistakes are seen as personal, reflect badly on the person—the person is seen as a mistake. Little time for learning”) and prompted participants to reflexively “shift” to a more community-based orientation rooted in decoloniality and Indigenous pedagogy, such as “Appreciation” (“Mistakes are valued as opportunities for learning. People verbally show their appreciation for each other”). Ultimately, the purpose of these reminders was to invite participants to consider their positionality as non-Native school staff in primarily AN communities and schools and collectively develop a more culturally-responsive approach to advancing Indigenous youth suicide prevention. Within a framework of multi-sector involvement, cultural responsiveness, and humility, teachers, school mental health professionals, administrators, students, families, and community partners can engage in adaptive interventions together (Clauss-Ehlers & Garagiola, 2023).

In this study, mixed effects regression models tested for significant differences in moderators and found that change slopes in *Wellness Self-Efficacy, Prevention Self-Efficacy, and Postvention Behaviors* were significantly stronger (more positive) in Cohort 1 compared with Cohort 2. Teachers (n = 86) improved their Interpersonal Support Giving behaviors significantly more than therapists (n = 9) from pre- to post. We found that school policies about suicide prevention moderate changes in Suicide Prevention Self-Efficacy, where change slopes were significantly stronger (more positive) compared to individuals’ schools with lower-rated policies.

When considering the differential impacts of PC CARES for different kinds of participants, analyses revealed that interpersonal support scores increased more for teachers than they did for therapists. These findings suggest that groups starting with fewer experiences in offering interpersonal support and less mental health training had more room to grow in this capacity. They also reflect several implications for school mental health practice. In rural, under-resourced schools, many teachers serve a mental health support role in unofficial capacities, while their primary role is educator. In the school districts where PC CARES At School was implemented, school counselors and mental health clinicians are regional, itinerant positions, where a given village or school has a provider present and in residence about 1–3 days per month.

PC CARES demonstrated particular value in schools with fewer prevention policies and support systems (Fig. 4). Participants from these schools reported limited existing protocols and practices, leaving substantial gaps in staff preparedness. This created greater opportunities for meaningful growth, as PC CARES’ flexible and easy to use model addresses critical prevention and postvention needs without relying on extensive policies. Given the rising number of youth mental health issues and suicidal behaviors, this finding underscores the urgent need for scalable interventions like PC CARES, especially for under-resourced schools where traditional suicide prevention frameworks are insufficient. It is worth noting that all outcome variables measured in this study improved from pre- to post-intervention despite only 1 or 2 attendees from some schools.

PC CARES invites participants to take action in whatever way feels comfortable for them. There is no requirement for participants to enact evidence-based prevention strategies (counseling on access to lethal means, for example), but participants likely get more comfortable providing the support that they already offer. The State of Alaska mandates suicide awareness and prevention training for school staff every two years. While PC CARES is an approved training, the commonly used training tool to fulfill this requirement is an asynchronous online module which educators complete alone and at their own pace. It is widely used [2683 eLearning modules completed in FY23 (Morrison, 2023)] but its efficacy is unknown.

Our results support the creation of policy that offers suicide prevention training to a diversity of different actors within school systems and communities, encouraging collaborative relationships to increase prevention actions. Given the key role of professional development for school and youth program staff, curricula that includes cultural responsiveness and humility is vital for positive outcomes for youth (Richmond et al., 2018).

Trusted adults in professions that position them in close, supportive contact with youth can benefit by obtaining continuing education credits or hours—which are required for license renewal in teaching and clinical practice—through participation in PC CARES. Training in PC CARES is thus easily built into the existing professional development infrastructures of these supportive community roles.

Particularly for complex interventions, scaling up may warrant rigorous research to determine how, when, and with whom to scale-up (Zamboni et al., 2019). Future iterations of PC CARES could focus on tailoring recruitment and training strategies to prioritize individuals with the most to gain, such as those with limited experience providing interpersonal support. A key challenge with scaling up is the tension between the need to establish the proof of concept and the need to design an innovation that would be fit-for-scale (Zamboni et al., 2019).

Targeting school staff and community members, this delivery of PC CARES aimed to change the school environment by changing how suicide is talked about, encouraging safety and mental health support for students both generally and after a suicide tragedy to reduce contagion, and encouraging participants to consider how they can take action on a school level (if they want to). Evaluation outcomes suggest that PC CARES is an effective intervention to increase suicide prevention and wellness self-efficacy, collaborative relationships (CoP) and importantly, enhancing universal and selective prevention-oriented behaviors among adults who interact with youth in their schools and communities.

## Limitations and Future Directions

This analysis should be considered with several limitations in mind. First, as a response to the COVID pandemic in 2020–2022, PC CARES was delivered online in partnership with remote schools. This shifted the demographics of learning circle participants from being primarily Alaska Native people from the region to including more White teachers from the lower 48 states. As a result, these findings may represent external service providers in the region and might be less generalizable to local AN residents.

We also note that, for purposes of interpretative clarity and theory building, we conducted multiple single-predictor and moderator analyses rather than adding many simultaneous covariates to a single set of models. This choice provides the desired interpretive clarity but carries the risk of false positive findings. Thus, moderator findings, particularly those found on only a single outcome, should be interpreted with some caution. Future research on larger samples will be needed to more comprehensively assess how all these variables work together in their association with the outcomes.

While the current study focused on the quantitative outcomes of the PC CARES At-School implementation, complementary qualitative data were also collected and analyzed to examine participants’ engagement, interpretation of the research content, and local relevance of suicide prevention strategies. Integrating the qualitative findings (Evans et al., 2026) with the quantitative results in a future mixed-methods analysis could deepen understanding of how PC CARES fosters change in school environments and inform adaptation and implementation across diverse educational contexts. Other future directions include linking this support-giving and increases in collaborative relationships to youth outcomes during the same time period as the intervention.

## Conclusion

Suicide remains a persistent and significant health inequity for rural Alaska Native (AN) youth, requiring innovative, community-based approaches. Findings from PC CARES At School demonstrate the effectiveness of a virtual, school-based intervention in enhancing participant knowledge, self-efficacy, and collaborative behaviors related to suicide prevention and mental health promotion. The program successfully engaged staff across diverse roles—including teachers, administrators, and counselors—in meaningful dialogue and actionable strategies for supporting student mental health. Notably, participants with less prior mental health training, interpersonal support experience, and those from schools with fewer suicide prevention policies in place experienced the most significant gains.

The study’s results underscore the importance of building communities of practice (CoP) within schools to foster collaboration and collective, self-determined action. These findings reinforce the theoretical foundation of PC CARES, which emphasizes collaboration and connection across varied school and community roles to address complex social and health challenges. By focusing on cultural humility and self-reflection, the program allowed participants to contextualize suicide prevention strategies to the unique realities of rural AN communities.

The policy and practice implications of this study are clear. Training school staff across diverse roles enhances their capacity to collaboratively address suicide prevention and mental health promotion, even in the absence of significant external resources. Moreover, the program’s effectiveness in a virtual format demonstrates its scalability and adaptability, providing a practical solution to mental health workforce shortages and structural disparities. Policymakers and education leaders should prioritize integrating culturally responsive, community-based programs like PC CARES into professional development curricula. These findings also demonstrate the potential of such interventions to serve as scalable models for addressing broader youth mental health challenges in similarly underserved regions. By investing in such programs, schools and communities can build systems of support, ultimately reducing the burden of suicide and fostering healthier, more resilient school environments for all students.
